# Supplementary figures and images for: Multi-insecticide resistant malaria vectors in the field remain susceptible to malathion, despite the presence of Ace1 point mutations
Source: PLoS Genet. 2022 Feb 10;18(2):e1009963. doi: 10.1371/journal.pgen.1009963 (PMC8830663; doi:10.1371/journal.pgen.1009963)

## Slide 1
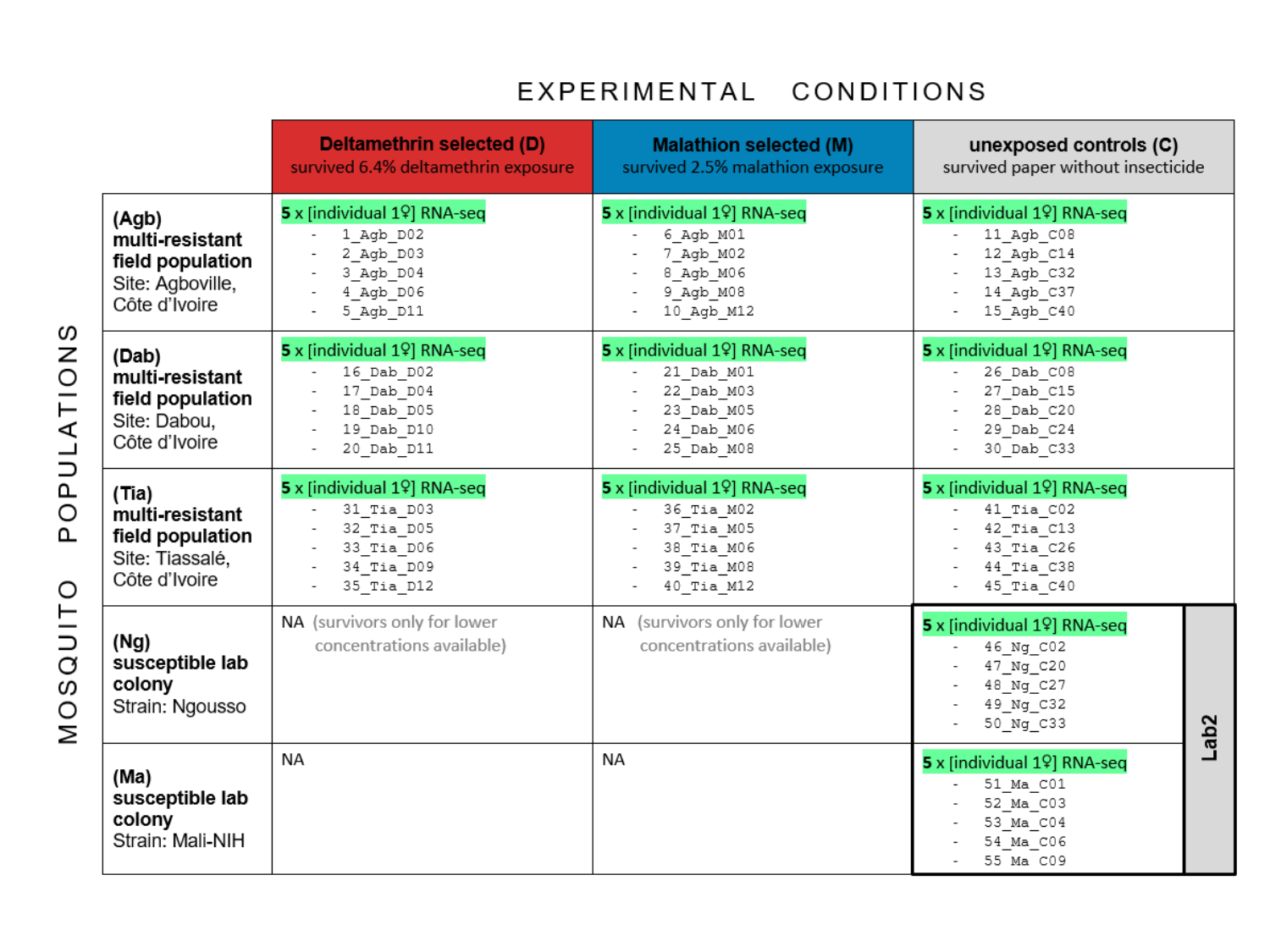

Supplement: S1 Fig — We sequenced five individual mosquitoes (i.e. five biological replicates) per An. coluzzii population and experimental condition. The five laboratory-reared An. coluzzii populations were three multi-resistant populations from southern Côte d’Ivoire collected at the larval stage in Agboville (Agb), Dabou (Dab) and Tiassalé (Tia) and two insecticide susceptible laboratory colonies Ngousso and Mali-NIH (Lab2). The three experimental conditions were insecticide-unexposed control (C), selected against 6.4% deltamethrin (D) and 2.5% malathion (M). One biological sample denotes RNA extracted from an individual, laboratory-reared, 3- to 6-day-old female An. coluzzii mosquito. Each mosquito was sequenced twice in different runs, producing two technical replicates. NA, not available. (PPTX) [file pgen.1009963.s001.pptx]

## Slide 1
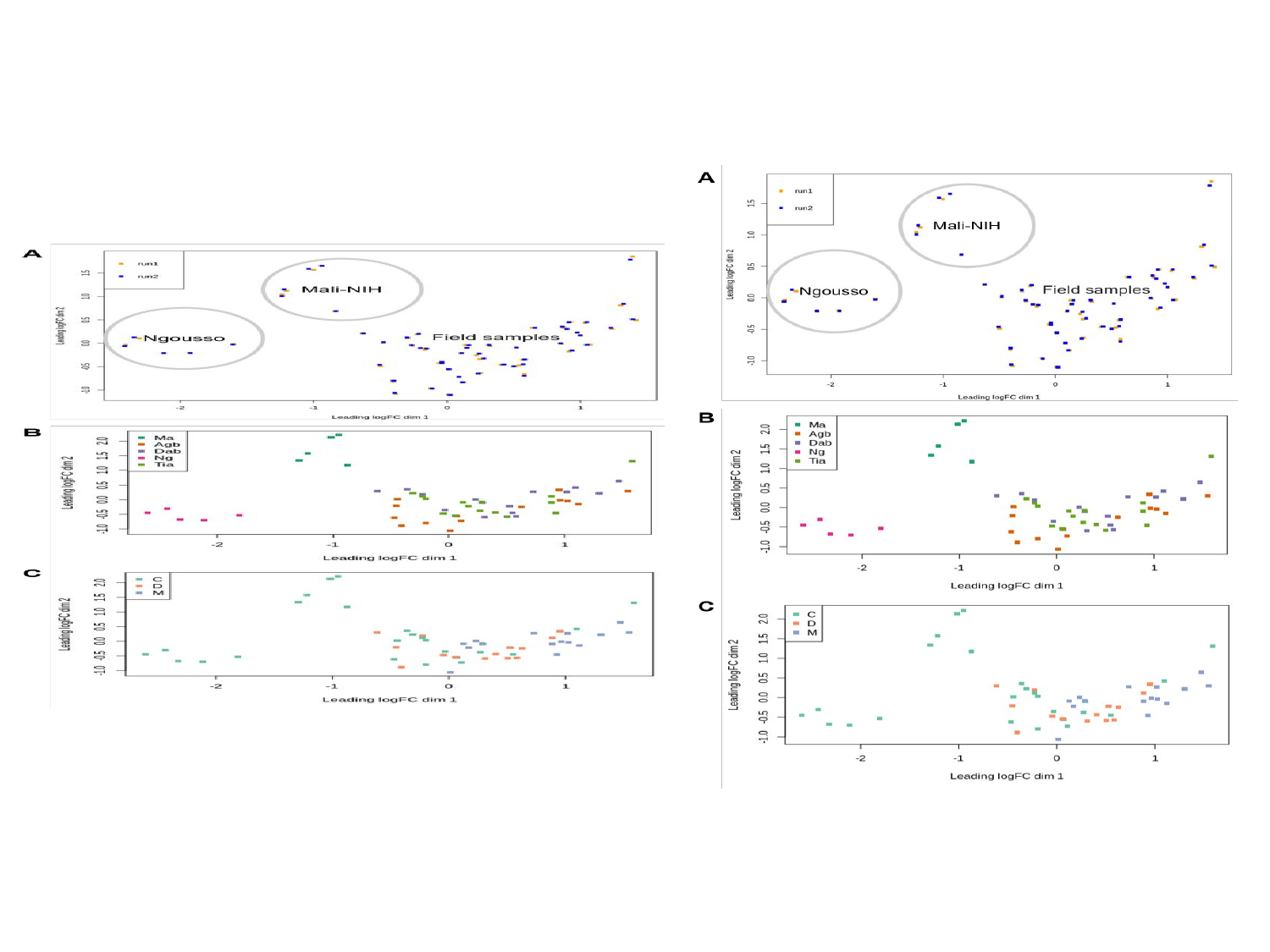

Supplement: S2 Fig — (A) MDS plot showing lower variation between technical replicates (yellow: run1 and blue: run2) than between biological replicates (labels removed for better visibility). (B) MDS plot using summed read counts of the two runs showing clear separation between two lab colonies and field populations (Agb, Dab, Tia) and also between the Mali-NIH and Ngousso lab colonies, but not between the three field populations. (C) MDS plot showing that there were no obvious patterns indicative of experimental condition: insecticide-unexposed control (C), selected against 6.4% deltamethrin (D), and 2.5% malathion (M). (PPTX) [file pgen.1009963.s002.pptx]

## Slide 1
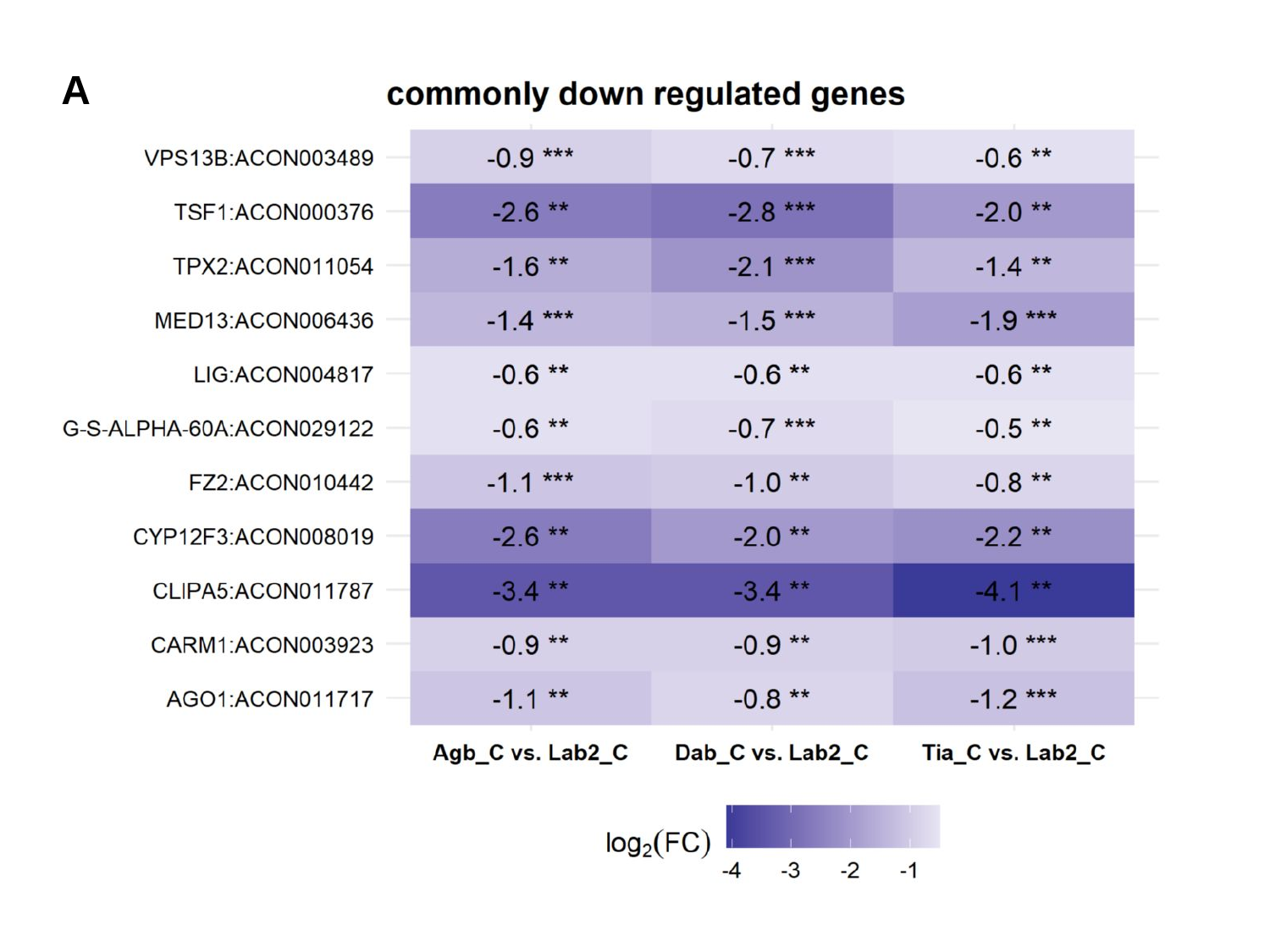

A

## Slide 2
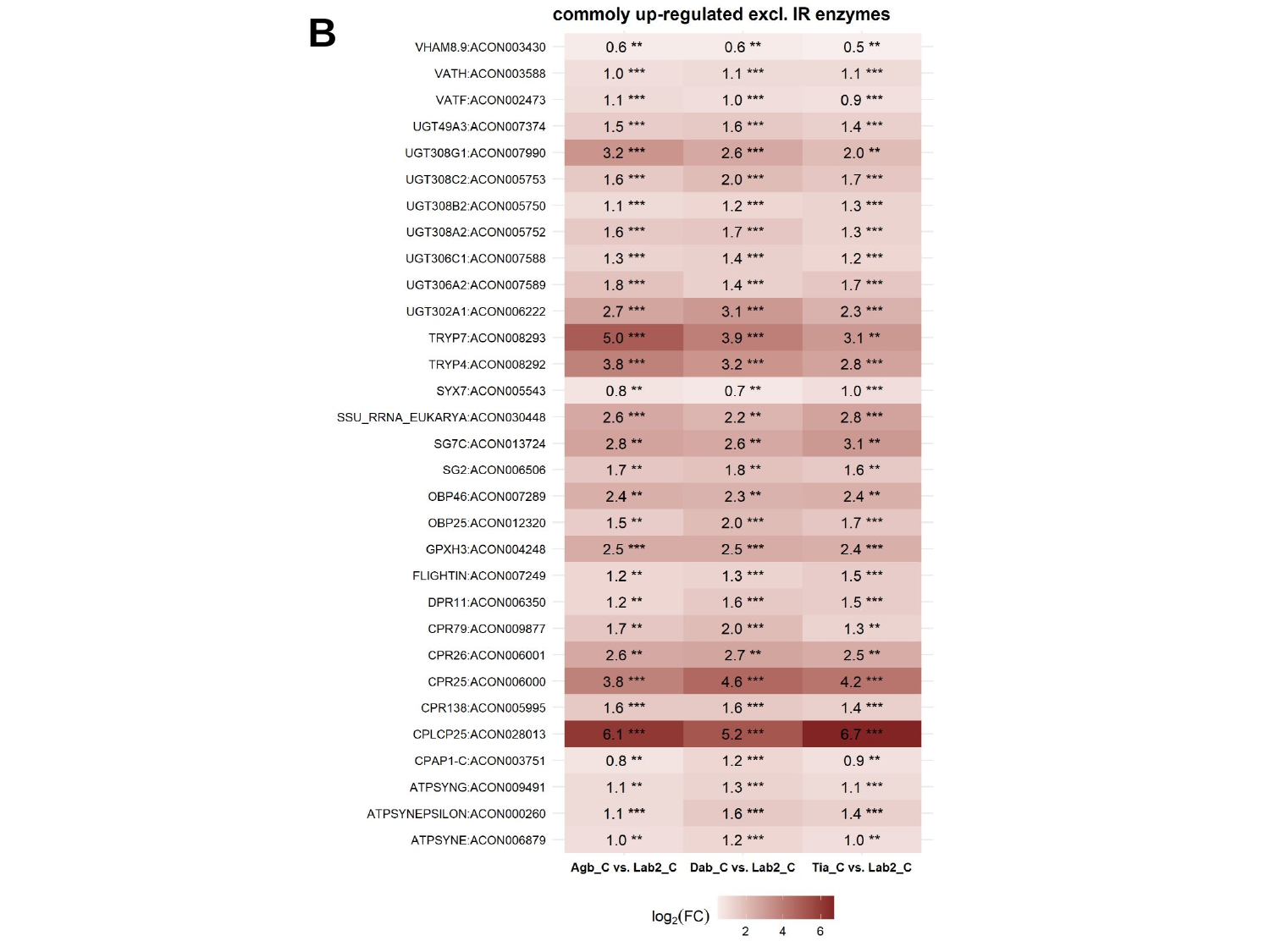

B

## Slide 3
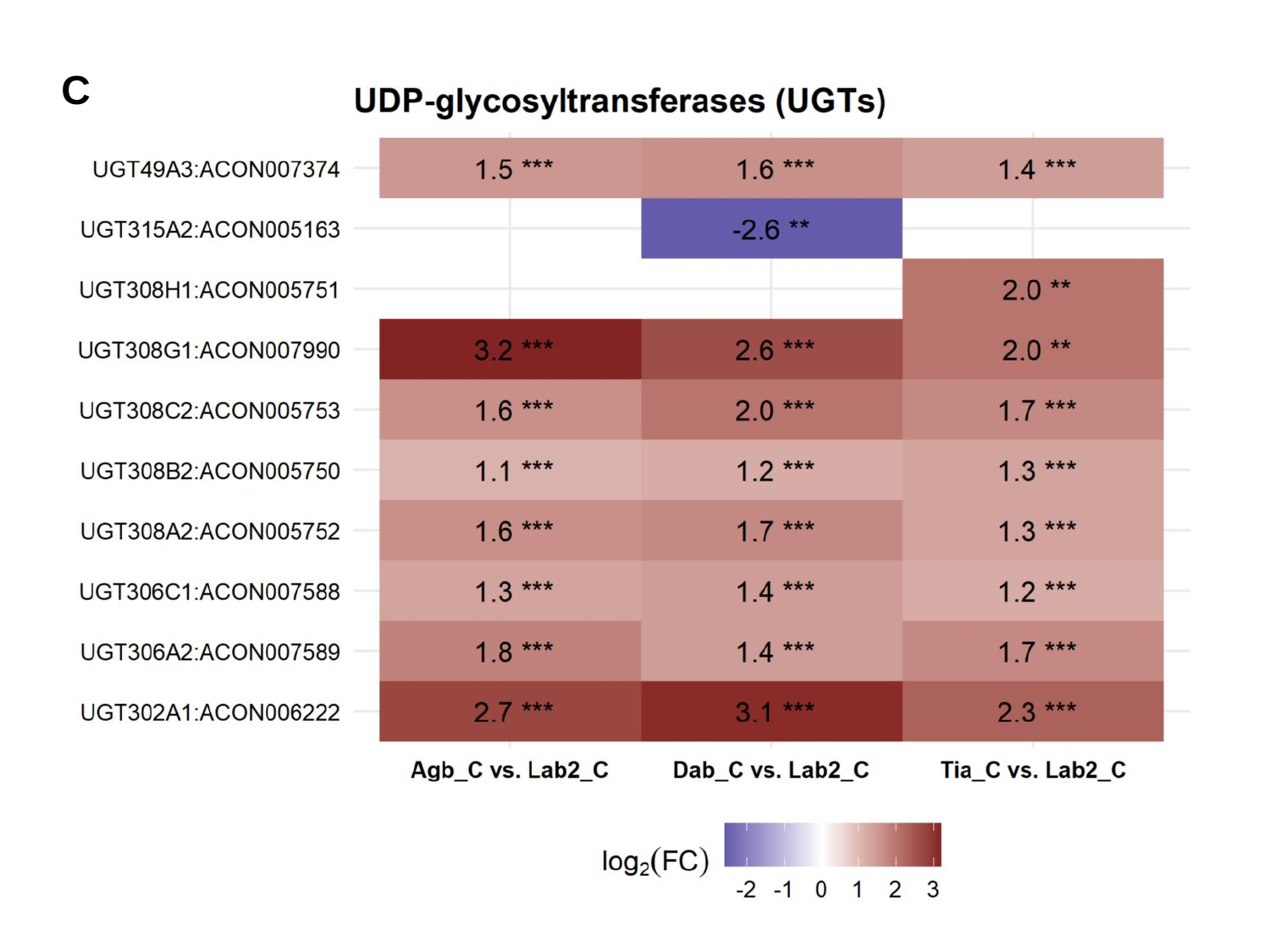

C

## Slide 4
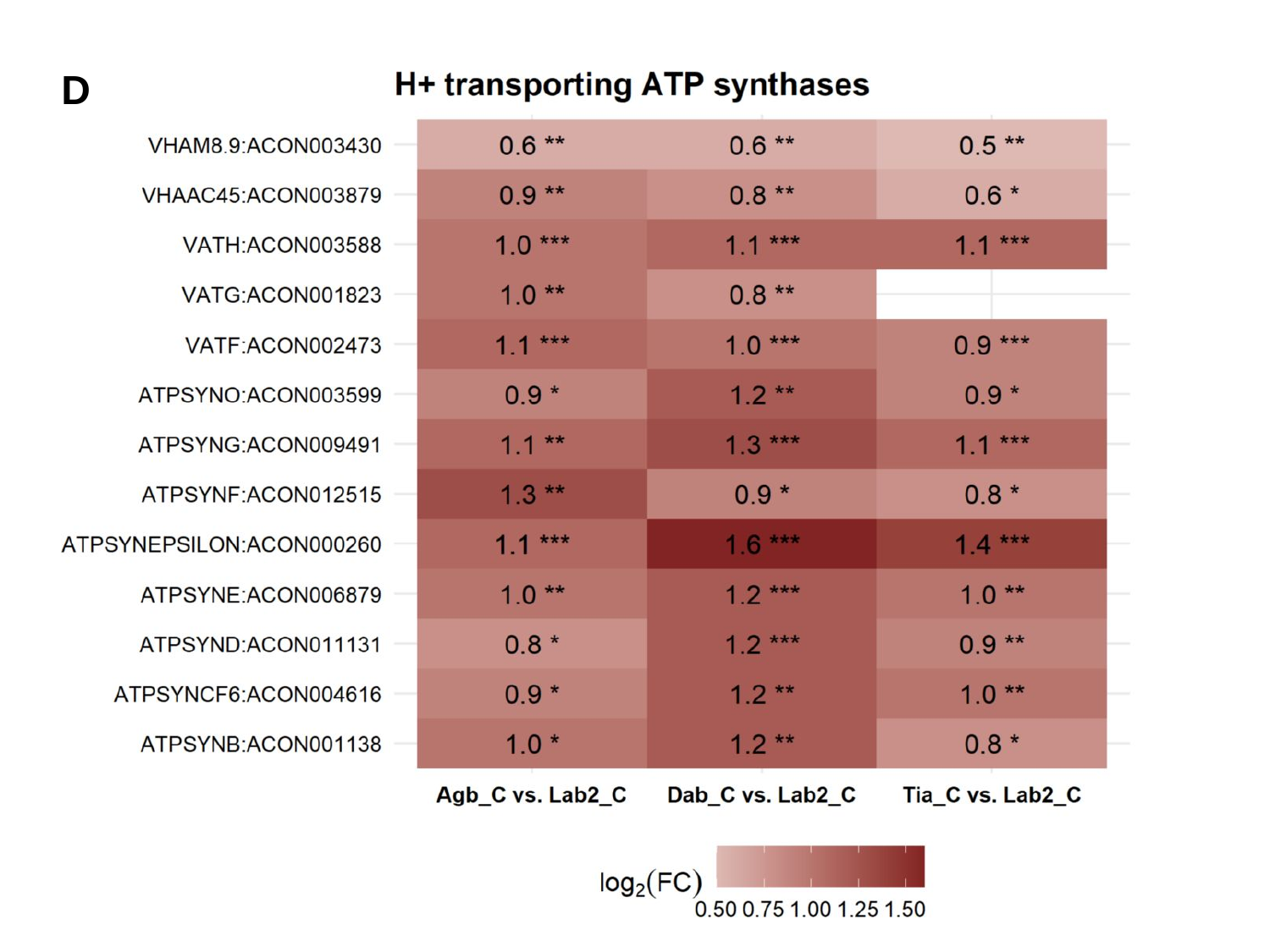

D

## Slide 5
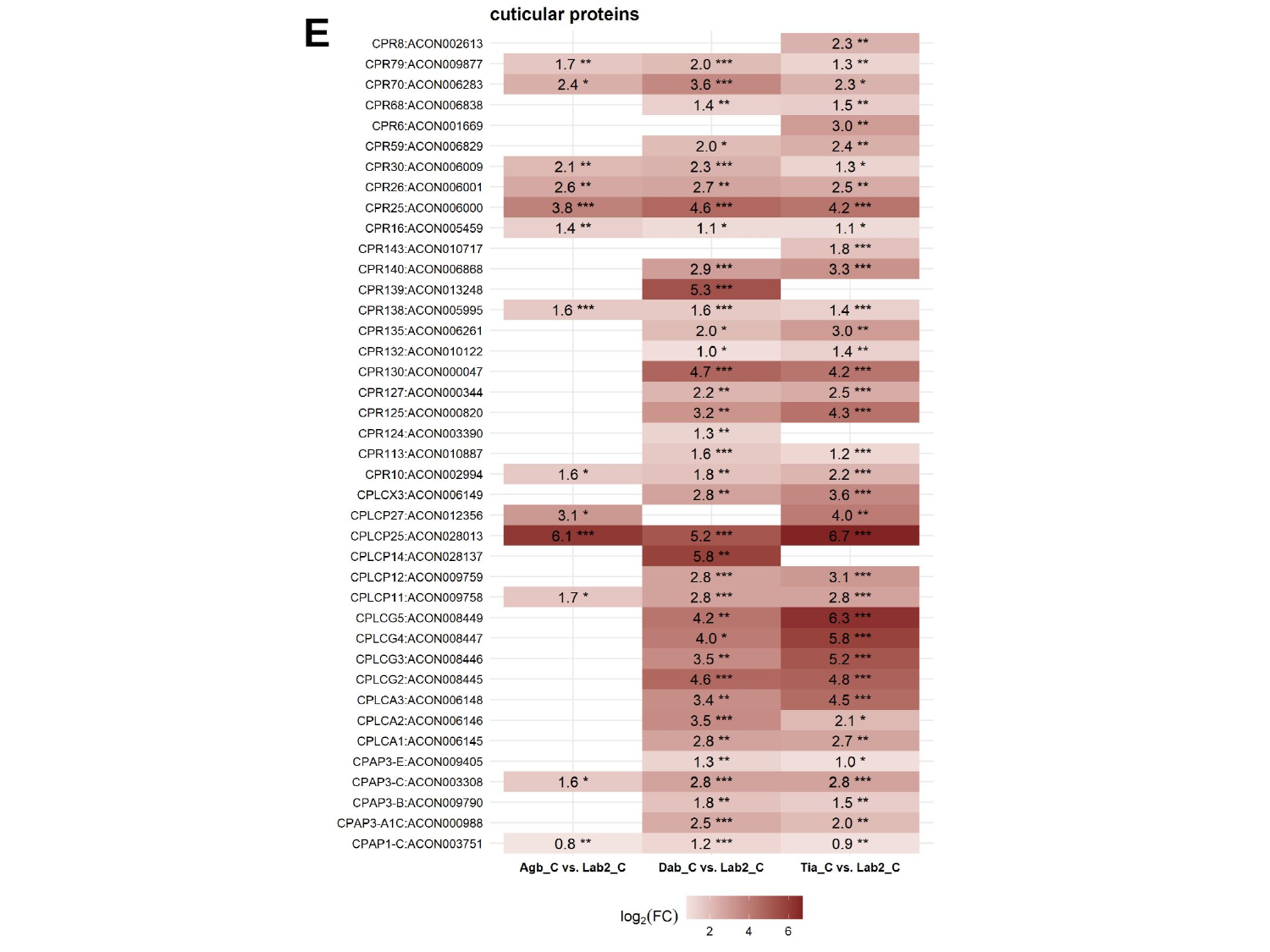

E

## Slide 6
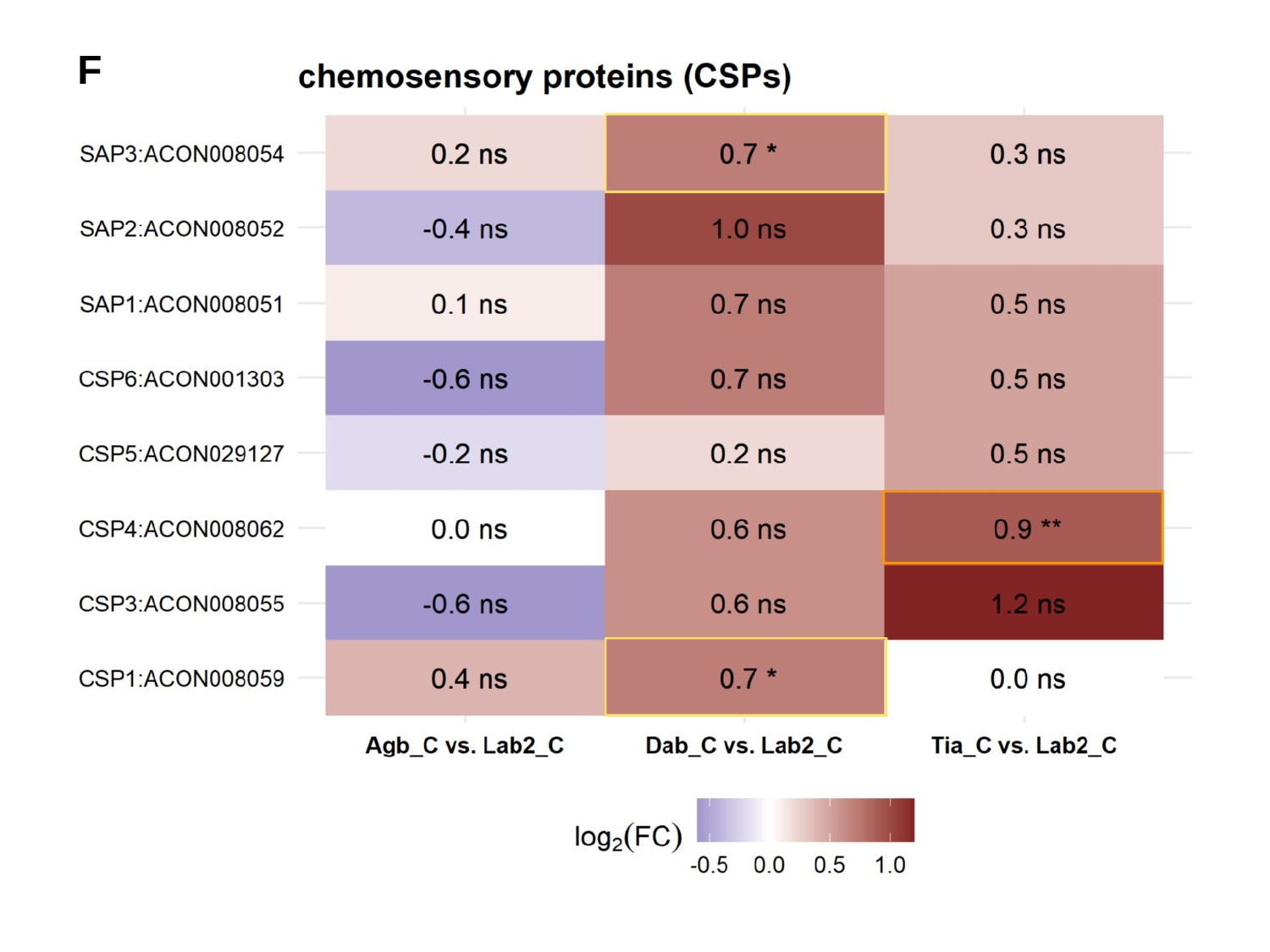

F

## Slide 7
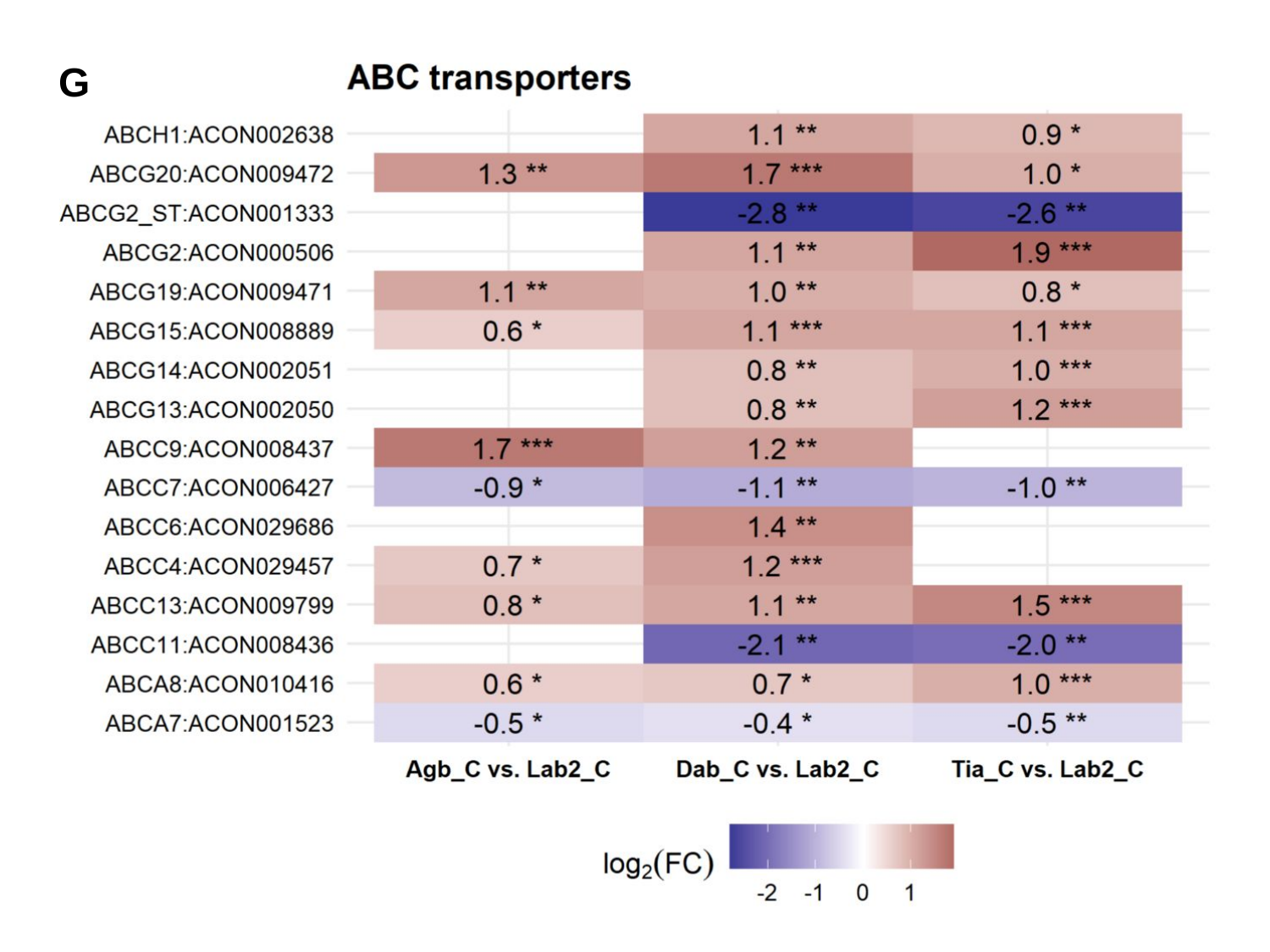

G

## Slide 8
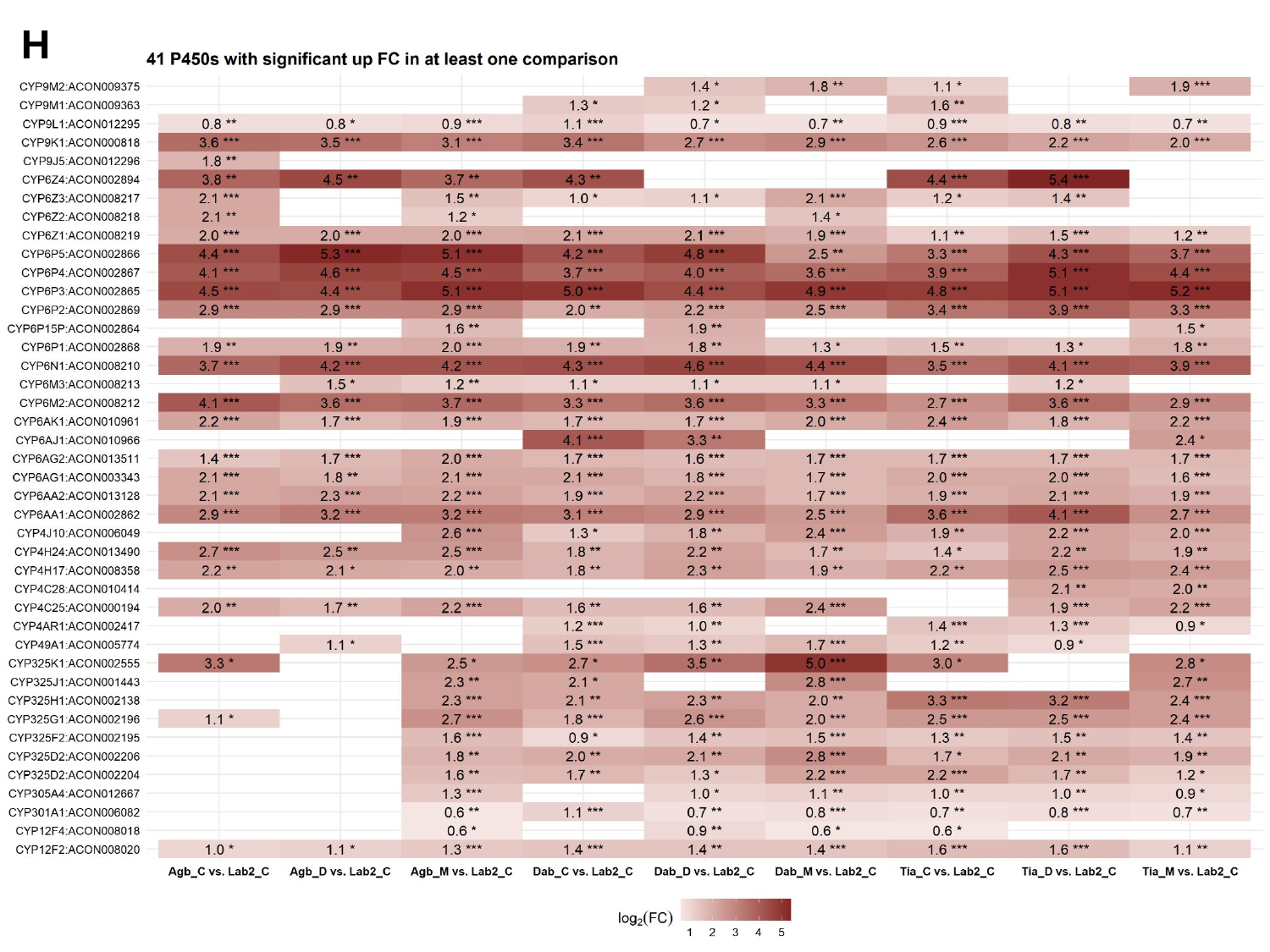

H

Supplement: S3 Fig — (A) Commonly underexpressed genes; (B) commonly overexpressed genes apart from the three major detoxification enzyme families shown in Fig 4; (C) Uridine diphosphate UDP-glycosyltransferases (UGTs); (D) H+-transporting ATP synthases; (E) cuticular proteins; (F) chemosensory proteins (CSPs); (G) ATP-binding cassette (ABC) transporters; and (H) 41 P450s, in addition to the unexposed controls also including the insecticide selected samples compared to the laboratory colonies. On the left of the heat map are the putative, orthology-derived gene names and ACON gene IDs for which **FDR ≤ 0.01 in at least one comparison (except for CSPs). The number displayed on the coloured tiles shows the log2 fold change (log2FC) with tiles in red depicting overexpression (log2FC > 0) and blue underexpression (log2FC < 0). Levels of significance *FDR ≤ 0.05; **FDR ≤ 0.01; and ***FDR ≤ 0.001. Tiles were left empty when FDR > 0.05, except for S3F Fig for CSPs includes FDR > 0.05, not significant (ns). (PPTX) [file pgen.1009963.s003.pptx]

## Slide 1
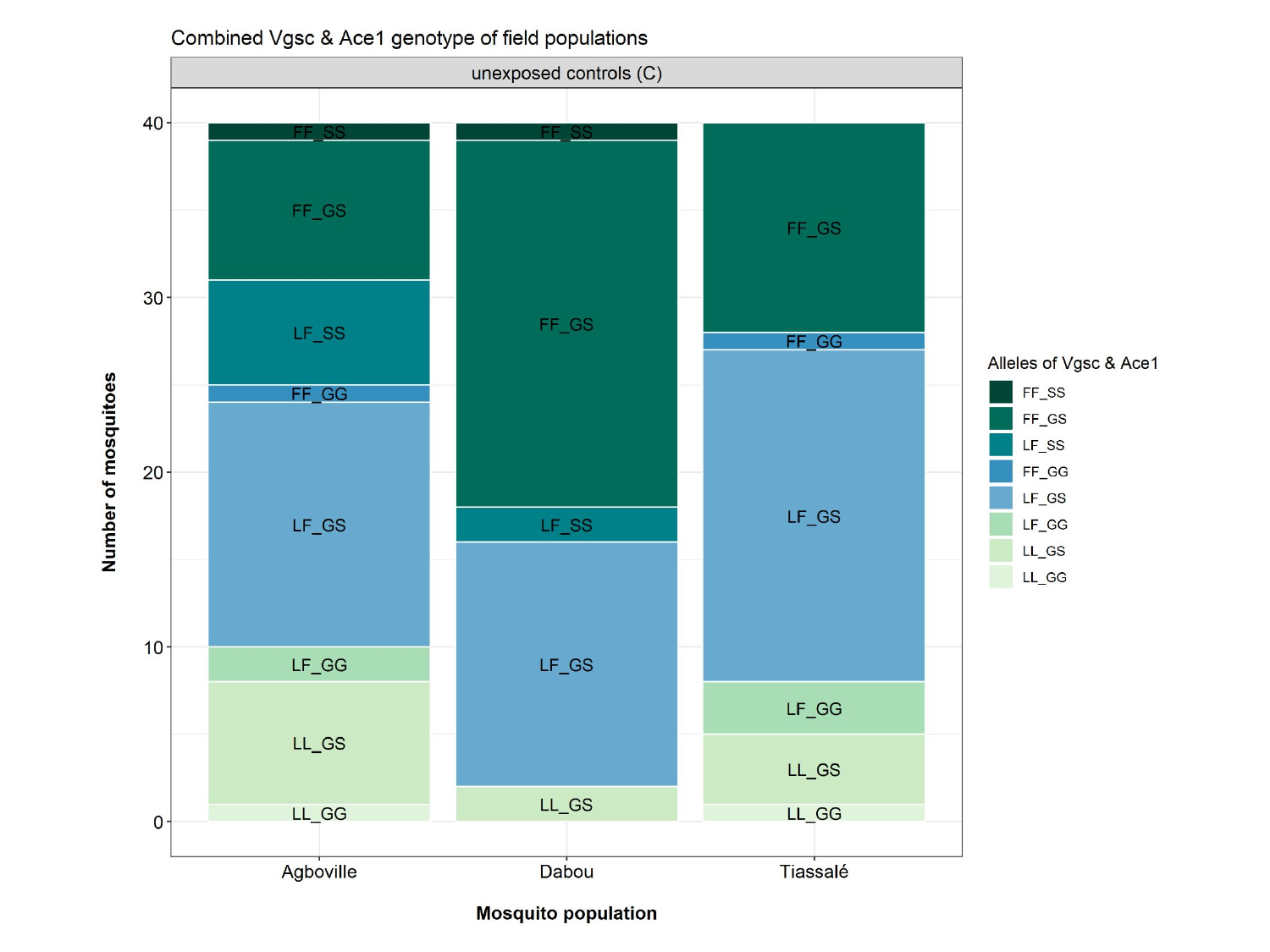

Supplement: S4 Fig — Bar plots showing the combined qPCR results on leg DNA for two target-site mutations (Vgsc-L995F and Ace1-G280S), i.e. in which combination the resistance-associated alleles occurred in 40 insecticide-unexposed individuals per field population. (PPTX) [file pgen.1009963.s004.pptx]
